# Medial nucleus accumbens dopamine receptors modulate motivation for wheel running in male mice

**DOI:** 10.1038/s41386-025-02136-w

**Published:** 2025-06-12

**Authors:** Naoya Nishitani, Taisuke Kokume, Harumi Taniguchi, Katsuyuki Kaneda

**Affiliations:** Laboratory of Molecular Pharmacology, Institute of Medical, Pharmaceutical and Health Sciences, Kanazawa University, Kanazawa, 920-1192 Japan

**Keywords:** Addiction, Motivation, Neurophysiology, Reward

## Abstract

Abnormal motivation for natural rewards is a hallmark of various psychiatric disorders, including behavioral addiction. The mesolimbic dopamine pathway has been identified as a critical modulator of motivated behavior primarily based on studies using food-reinforced operant tasks. However, the focus on food rewards in previous studies limits the generalizability of these findings to other natural rewards implicated in behavioral addiction. In this study, we investigated the reinforcing and high motivational properties of wheel running in rodents by developing a wheel running-reinforced operant conditioning procedure. This procedure allowed for the independent quantification of appetitive and consummatory behaviors as operant responses and running duration, respectively, facilitating an in-depth exploration of the role of dopamine signaling in the medial nucleus accumbens (mNAc) in wheel running motivation. The results indicated that the systemic inhibition of dopamine D_1_ and D_2_ receptors suppressed appetitive behavior, whereas inhibition of D_1_ receptors reduced consummatory behavior. Similarly, inhibition of mNAc neural activity and blockade of D_1_ and D_2_ receptors within this region diminished appetitive behavior, with D_1_ receptor inhibition uniquely impairing consummatory behavior. Fiber photometry recordings demonstrated that decreases in mNAc neural activity and increases in dopamine levels preceded appetitive behavior. Additionally, mNAc neural activity and dopamine levels were elevated following cues signaling the availability of wheel running. Furthermore, systemic D_1_ receptor inhibition attenuated the reduction in mNAc neural activity observed during appetitive behavior. These findings suggest that increased dopamine release and the subsequent D_1_ receptor-mediated suppression of mNAc neural activity underlie the motivated behavior for wheel running.

## Introduction

Motivation for rewarding stimuli is a fundamental process across species, including humans. However, excessive motivation for rewards, such as drugs or natural stimuli like food, can lead to substance use disorder or compulsive food seeking [1–4]. Moreover, physical activity, specifically wheel running in rodents, is also considered rewarding. Previous studies have demonstrated that rodents develop conditioned place preference (CPP) for wheel running [5–7], learn to lever press or nose poke to access running wheels [8–10], and are more strongly reinforced by wheel running than by palatable food [10]. Furthermore, wheel running in rodents shares several characteristics with rodent models of substance use disorders [11, 12]; chronic wheel running induces aggressive behavior in rats when deprived of running wheel access [13] and increases the α-amino-3-hydroxy-5-methyl-4-isoxazolepropionic acid/N-methyl-D-aspartate current ratio at synapses in the ventral tegmental area (VTA) dopaminergic neurons [14]. Collectively, these findings suggest that wheel running in rodents has intrinsically high motivational properties similar to those of addictive drugs.

Accumulating evidence suggests the involvement of the mesolimbic dopamine system in motivation for both drugs and natural rewards [15–17]. The nucleus accumbens (NAc), which receives dense dopaminergic projections from the VTA, plays a central role in reward processing [18, 19]. Several studies, including ours, have demonstrated that NAc neuronal activation is associated with voluntary wheel running [20–23]. For instance, ΔFosB, a marker of neuronal activation, accumulates in a subset of NAc neurons following chronic wheel running, and overexpression of ΔFosB in the NAc increases wheel running distance in rats [20]. Additionally, we previously demonstrated that c-Fos, another marker of neuronal activity, shows increased expression in the medial NAc (mNAc) during chronic wheel running or when mice with prior wheel running experience are exposed to but prevented from running on a running wheel [23]. Dopamine signaling has also been involved in wheel running behavior [10, 24, 25]. Blocking dopamine D_1_ and D_2_ receptors reduces voluntary wheel running [24]. VTA dopaminergic neurons exhibit burst firing at the onset and offset of running [25], and their firing rate increases in mice trained to nose poke for wheel access [10]. Motivated behavior for rewarding stimuli can be conceptualized into two distinct components: appetitive (reward-seeking) and consummatory (reward-taking) [26–28]. However, in voluntary paradigms, it is challenging to quantify these components independently in wheel running behavior.

Operant conditioning tasks offer a reliable method for analyzing appetitive and consummatory components of reward. Typically, lever presses or nose pokes gauge the appetitive aspect, while the consumed reward volume (e.g., food or drugs) indicates the consummatory facet [29, 30]. Although extensively used to explore the neural basis of motivation for natural rewards through food-reinforced tasks [31–33], variations in reward processing question the applicability of these findings to other rewards like wheel running [3, 34, 35]. Thus, exploring the neural basis of motivation for wheel running is crucial for a comprehensive understanding of natural reward systems, essential for developing behavioral addiction treatments.

To address this gap, we investigated the roles of mNAc neural activity and dopamine signaling in wheel running motivation, by developing an operant conditioning procedure reinforced by wheel running. In this procedure, mice could run on a wheel after completing a predetermined number of nose pokes, enabling separate quantification of appetitive and consummatory components of motivation. The number of nose pokes and duration of wheel running were interpreted as indicators of appetitive and consummatory behaviors, respectively. We then examined whether inhibiting neural activity or dopamine signaling in the mNAc affects motivation for wheel running. Additionally, we employed fiber photometry to measure neural activity and dopamine release in the mNAc and analyzed their relationship during appetitive and consummatory behaviors.

## Materials and methods

### Animals

**Fig. 1:**
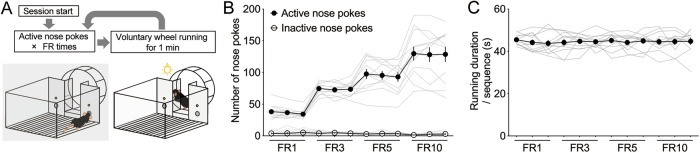
Establishment of a behavioral procedure to quantify the appetitive and consummatory components of motivation for wheel running. **A** Schematic representation of the operant wheel running task. The number of nose pokes **B** and the duration of wheel running **C** in sessions meeting the training criteria (n = 12). Data are expressed as means ± SEM.

**Fig. 2:**
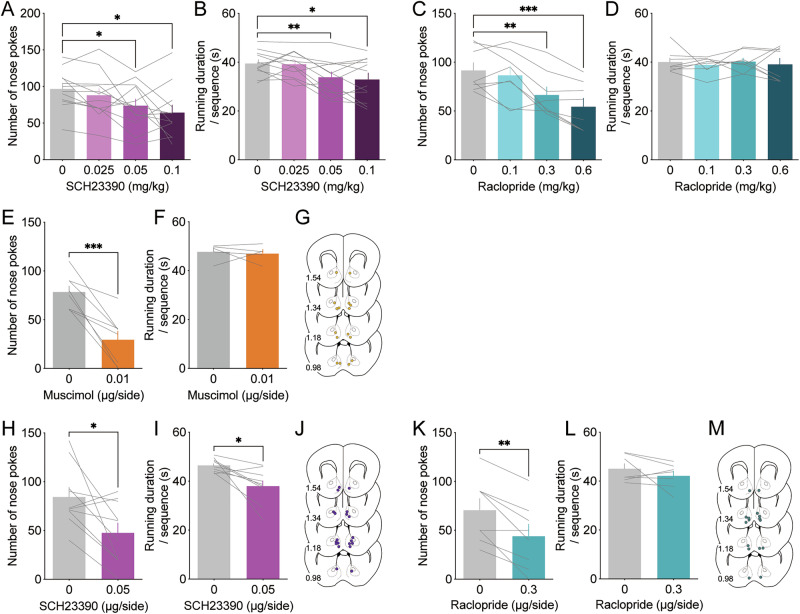
Effect of dopamine D_1_ and D_2_ receptor inhibition in the mNAc on motivated behavior for wheel running. Effect of the D_1_ receptor antagonist SCH23390 on the number of nose pokes **A** and the duration of wheel running **B** in the FR10 schedule (n = 12; *P < 0.05, **P < 0.01). Effect of the D_2_ receptor antagonist raclopride on the number of nose pokes **C** and the duration of wheel running **D** in the FR10 schedule (n = 9; **P < 0.01, ***P < 0.001). Effect of intra-mNAc muscimol infusion on the number of nose pokes **E** and the duration of wheel running **F** in the FR10 schedule (n = 5–8; ***P < 0.001). **G** Cannula tip placements for muscimol infusion. Effect of intra-mNAc SCH23390 infusion on the number of nose pokes **H** and the duration of wheel running **I** in the FR10 schedule (n = 9–10; *P < 0.05). **J** Cannula tip placements for SCH23390 infusion. Effect of intra-mNAc raclopride infusion on the number of nose pokes **K** and the duration of wheel running **L** in the FR10 schedule (n = 7–8; **P < 0.01). **M** Cannula tip placements of raclopride infusion. Data are expressed as means ± SEM.

**Fig. 3:**
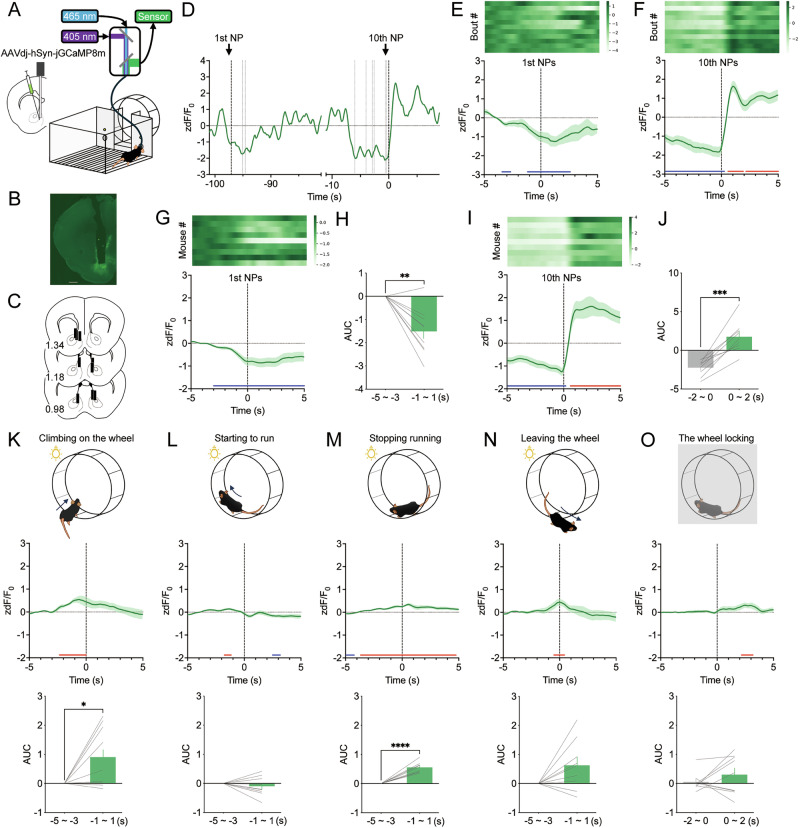
Neural activity in the mNAc during the operant wheel running task. **A** Schematic of the fiber photometry setup. Representative images of AAVdj-hSyn-GCaMP8m injection **B** and optic fiber implantation **C** into the mNAc. Numbers indicate approximate anteroposterior distance (mm) from bregma. Scale bar = 500 µm. **D** Representative GCaMP signals during the operant wheel running task. **E** Heatmap (top) and averaged GCaMP signals (bottom) around the first nose pokes of one mouse (n = 11 bouts). **F** Heatmap (top) and averaged GCaMP signals (bottom) around the tenth nose pokes of one mouse (n = 11 bouts). **G** Heatmap (top) and mean GCaMP signals (bottom) around the first nose pokes (n = 9). **H** AUC of mean GCaMP signals from −5 s to −3 s and −1 s to 1 s aligned with the first nose pokes in the FR10 schedule (n = 9; **P < 0.01). **I** Heatmap (top) and mean GCaMP signals (bottom) around the tenth nose pokes (n = 9). **J** AUC of mean GCaMP signals from −2 s to 0 s and 0 s to 2 s aligned with the tenth nose pokes in the FR10 schedule (n = 9; ***P < 0.001). Mean GCaMP signals and AUC of mean GCaMP signals from −5 s to −3 s and −1 s to 1 s aligned with the onset of climbing on the wheel **K**, starting to run on the wheel **L**, stopping running on the wheel **M**, leaving the wheel **N**, and AUC of mean GCaMP signals from −2 s to 0 s and 0 s to 2 s aligned with the onset of the wheel locking **O** from nine mice (n = 9; *P < 0.05, ****P < 0.0001). Vertical dashed lines indicate the onset of the event, and horizontal dashed lines indicate the baseline. Blue and red lines indicate statistically significant decreases and increases from 0, respectively (bootstrap test, higher bound 95% extended bootstrapped CI < 0 and lower bound 95% extended bootstrapped CI > 0, respectively). Data are expressed as means ± SEM.

**Fig. 4:**
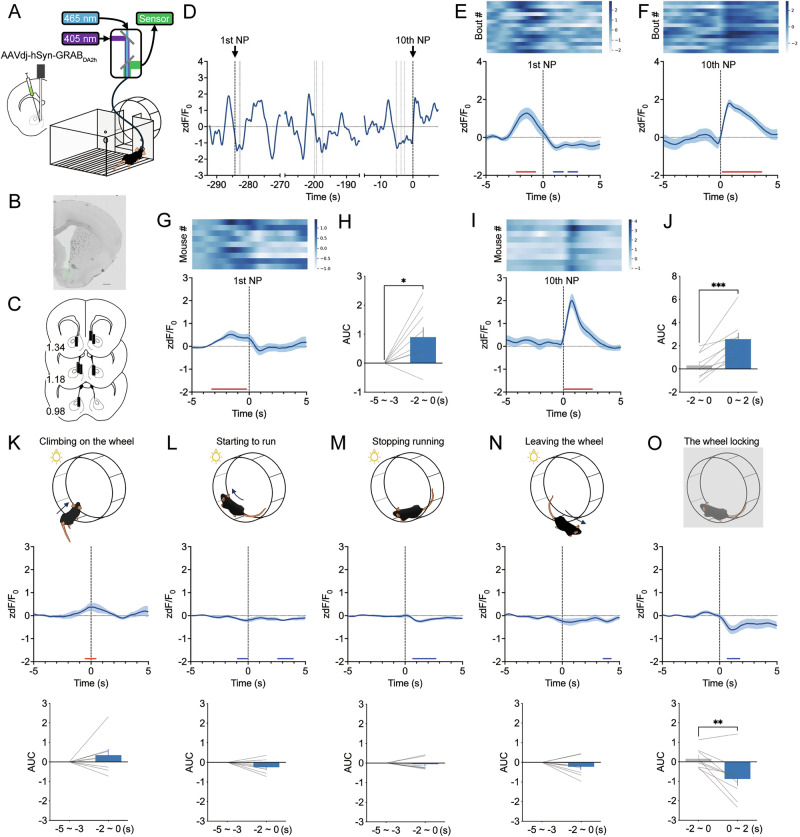
Dopamine release in the mNAc during the operant wheel running task. **A** Schematic representation of the fiber photometry setup. AAVdj-hSyn-GRAB_DA2h_ injection **B** and optic fiber implantation **C** into the mNAc. Numbers indicate the approximate anteroposterior distance (mm) from bregma. Scale bar = 500 µm. **D** Representative GRAB_DA2h_ signals from one mouse during the operant wheel running task. **E** Heatmap (top) and averaged GRAB_DA2h_ signals (bottom) around the first nose pokes of one mouse (n = 14 bouts). **F** Heatmap (top) and averaged GRAB_DA2h_ signals (bottom) around the tenth nose pokes of one mouse (n = 14 bouts). **G** Heatmap (top) and mean GRAB_DA2h_ signals (bottom) and around the first nose pokes (n = 9). **H** AUC of mean GRAB_DA2h_ signals from −5 s to −3 s and −2 s to 0 s aligned with the first nose pokes in the FR10 schedule (n = 9; *P < 0.05). **I** Heatmap (top) and mean GRAB_DA2h_ signals (bottom) around the tenth nose pokes (n = 6). **J** AUC of mean GRAB_DA2h_ signals from −2 s to 0 s and 0 s to 2 s aligned with the tenth nose pokes in the FR10 schedule (n = 9; ***P < 0.001). Mean GRAB_DA2h_ signals and AUC of mean GRAB_DA2h_ signals from −5 s to −3 s and −2 s to 0 s aligned with the onset of climbing on the wheel **K** starting to run on the wheel **L**, stopping running on the wheel **M**, leaving the wheel **N**, and AUC of mean GRAB_DA2h_ signals from −2 s to 0 s and 0 s to 2 s aligned with the onset of the wheel locking **O** from nine mice (n = 9; **P < 0.01). Vertical dashed lines indicate the onset of the event, and horizontal dashed lines indicate the baseline. Blue and red lines indicate statistically significant decreases and increases from 0, respectively (bootstrap test, higher bound 95% extended bootstrapped CI < 0, and lower bound 95% extended bootstrapped CI > 0). Data are expressed as means ± SEM.

**Fig. 5:**
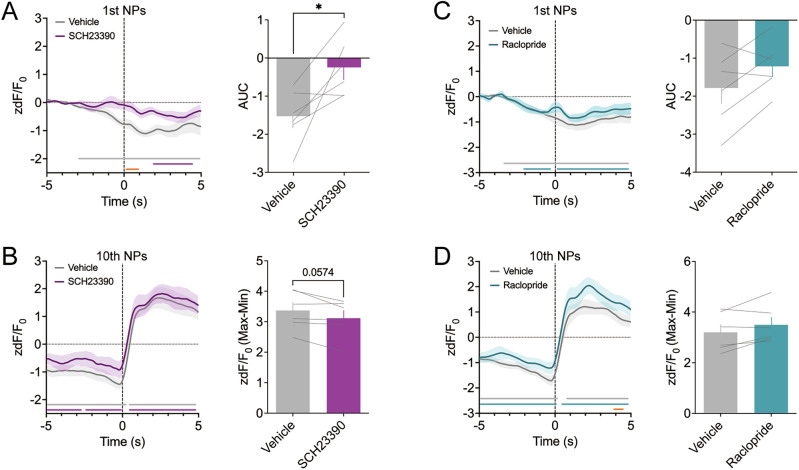
Effect of systemic administration of dopamine antagonists on neural activity in the mNAc during the operant wheel running task. **A** Mean GCaMP signals (left) and AUC of mean GCaMP signals from −1 s to 1 s (right) aligned with the first nose pokes in the FR10 schedule in vehicle- and SCH23390 (0.05 mg/ kg)-treated mice (n = 6; *P < 0.05). **B** Mean GCaMP signals (left) and rate of increase of mean GCaMP signals from minimum to maximum (right) aligned with the tenth nose poke in the FR10 schedule in vehicle- and SCH23390-treated mice. (n = 6). Vertical dashed lines indicate the onset of the event and horizontal dashed lines indicate baseline. Gray and magenta lines indicate statistically significant differences from 0 (bootstrap test, higher bound 95% extended bootstrapped CI < 0 or lower bound 95% extended bootstrapped CI > 0; gray: vehicle, magenta: SCH23390). **C** Mean GCaMP signals (left) and AU**C** of mean GCaMP signals from −1 s to 1 s (right) aligned with the first nose pokes in the FR10 schedule in vehicle- and raclopride (0.3 mg/kg)-treated mice (n = 6). **D** Mean GCaMP signals (left) and increase rate of mean GCaMP signals from minimum to maximum (right) aligned with the tenth nose poke in the FR10 schedule in vehicle- and raclopride-treated mice. (n = 6). Vertical dashed lines indicate the onset of the event, and horizontal dashed lines indicate the baseline. Gray and cyan lines indicate statistically significant differences from 0 (bootstrap test, higher bound 95% extended bootstrapped CI < 0 or lower bound 95% extended bootstrapped CI > 0; gray: vehicle, cyan: raclopride). Orange lines indicate a statistically significant difference between the zdF/F_0_ of vehicle-treated and SCH23390- or raclopride-treated mice (permutation test). Data are expressed as means ± SEM.

Male C57BL/6JJmsSlc mice (>7 weeks of age, n = 91) were utilized for experiments. Data from mice with incorrect infusion placements (n = 9) or incorrect fiber placements (n = 6) in the brain were excluded from the analysis. Different groups of mice were used in the experiments shown in Figs. 1, 2, and 4, whereas six of the nine mice used in the experiments shown in Fig. 3 were used in the experiments shown in Fig. 5 (Table 1). During breeding and experiments, the room temperature was kept stable at 22  ±  2 °C under a 12-h light/dark cycle. Mice could access food and water ad libitum. This study was conducted in accordance with the Institutional Animal Care and Use Committee guidelines at Kanazawa University (Approval No.: AP-204167). All efforts were made to minimize the suffering and number of mice used in this study.

**Table 1:** Summary of mice used in each experiment.

Figure	Group	Number of mice
Fig. 1B, C	Group 1	n = 12
Fig. 2A, B	Group 2	n = 12
Fig. 2C, D	Group 3	n = 8
Fig. 2E–G	Group 4	n = 8
Fig. 2H–J	Group 5	n = 10
Fig. 2K–M	Group 6	n = 8
Fig. 3	Group 7	n = 9
Fig. 4	Group 8	n = 9
Fig. 5	Group 7	n = 6
Fig. S2	Group 1	n = 12
Fig. S3A, B	Group 2	n = 12
Fig. S3C, D	Group 3	n = 8
Fig. S4	Group 1 (without fiber) Group 7 (with fiber)	n = 12 n = 9
Fig. S5	Group 7	n = 9
Fig. S6	Group 7	n = 9
Fig. S7	Group 7	n = 6
Fig. S8	Group 7	n = 6
Fig. S9	Group 7	n = 6

### Drugs

SCH23390 hydrochloride (Cayman Chemical, Ann Arbor, MI, USA), raclopride (Tokyo Chemical Industry, Tokyo, Japan), and muscimol (Wako, Osaka, Japan) were dissolved in saline and stored at −30 °C. All stock solutions were diluted with saline just before use.

### Production of adeno-associated virus (AAV) vectors

AAVs were prepared according to the previous report with slight modifications [36–38]. The details of AVV production are described in Supplementary Materials and Methods.

### Operant wheel running task

#### Behavioral setup

Experiments were conducted in a custom-built operant chamber with two nose poke ports and a running wheel (Fig. S1). The wheel was locked by a brake pad and unlocked in accordance with experimental conditions. A white cue light located above the wheel was turned on when the wheel was unlocked and turned off when the wheel was locked. A custom program running on an Arduino Uno and an Arduino Nano microcontroller (Arduino, Ivrea, Italy) was used to control the experimental logic.

#### Training

First, mice were habituated to the chamber and allowed to run freely on the wheel for 60 min. Second, mice were trained on a fixed ratio (FR) 1 schedule (FR1), in which the wheel was unlocked for 1 min immediately after mice made a nose poke to the active nose poke port. After the mice met the criteria (see Supplementary Materials and Methods), they moved on to the FR3 task, followed by the FR5 and FR10 tasks. Systemic drug injection, intra-mNAc drug infusion, and fiber photometry recordings were conducted in the FR10 task after they met the criteria. The details of the operant wheel running task are described in Supplementary Materials and Methods.

### Stereotaxic surgeries

The details of stereotaxic surgeries are described in Supplementary Materials and Methods.

### Fiber photometry

Fiber photometry recordings were performed in the operant chamber (Fig. S1) according to the previous report with slight modifications [38]. The zdF/F_0_ was temporally aligned with the onset of each event, which were then extracted and normalized by subtracting the pre-event baseline (the first and tenth nose pokes, average values from −5 s to −3 s of the first nose pokes; other events, average values from −5 s to −3 s of each event) for further analysis. To quantify the response magnitude, the area under the curve (AUC) of zdF/F_0_ was calculated by trapezoidal numerical integration across a fixed time scale. The details of fiber photometry analysis are described in Supplementary Materials and Methods.

### Histology

The details of histological analysis are described in Supplementary Materials and Methods.

### Statistical analysis

Data are expressed as means ± SEM. Data were analyzed using paired *t*-test or one-way repeated-measures analysis of variance (ANOVA) with Tukey post hoc test, two-way repeated-measures ANOVA with the Dunnett’s *post hoc* test using GraphPad Prism 9 software (GraphPad Software, La Jolla, CA, USA). In the presence of missing values, data were analyzed using a mixed-effects model fitted with Restricted Maximum Likelihood (REML) estimation instead of two-way repeated-measures ANOVA using GraphPad Prism 9. Differences with *P* < 0.05 were considered statistically significant. In the fiber photometry experiments, bootstrap and permutation tests were used to assess the differences in fluorescent signals from baseline and control groups, respectively [39], using a homemade python script. The details of statistical analysis are described in Supplementary Materials and Methods.

## Results

### Dopamine signaling via D_1_ and D_2_ receptors and neural activity in the mNAc are involved in motivation for wheel running

We developed an operant task reinforced by wheel running, where mice were required to perform a predetermined number of nose pokes to unlock the wheel for 1 min (Fig. 1A). During training, mice quickly learned to nose poke to unlock the wheel (Fig. S2). The number of nose pokes increased in proportion to the FR schedule (Fig. 1B), demonstrating the successful acquisition of appetitive behavior for wheel running. In contrast, the duration of wheel running during the 1-min unlocked period did not change significantly (Fig. 1C). These results suggested that once the mice gained access to the wheel, the consummatory behavior, i.e., wheel running, remained consistent across varying FR requirements.

Next, we evaluated the role of dopamine receptors in motivation for wheel running by examining the effects of systemic dopamine receptor blockade. The D_1_-like receptor antagonist SCH23390 (0.025–0.1 mg/kg) reduced both the number of nose pokes and the duration of wheel running in the FR10 task in a dose-dependent manner (Fig. 2A, B; nose pokes: ANOVA, *F*_2.183, 24.01_ = 5.554, *P* = 0.0089; post hoc test, 0 vs 0.025 *P* = 0.3564, 0 vs 0.05 *P* = 0.0309, 0 vs 0.1 *P* = 0.0414; duration: ANOVA, *F*_2.736, 30.10_ = 9.302, *P* = 0.0002; post hoc test, 0 vs 0.025 *P* = 0.9964, 0 vs 0.05 *P* = 0.0097, 0 vs 0.1 *P* = 0.0126, one-way repeated measures ANOVA with Tukey post hoc test). Session-level analysis revealed slight, but not significant, time-dependent changes in the number of nose pokes within single sessions (Fig. S3A, B). Conversely, the D_2_-like receptor antagonist raclopride (0.1–0.6 mg/kg) dose-dependently reduced the number of nose pokes without affecting the duration of wheel running (Fig. 2C, D; nose pokes: ANOVA, *F*_1.852, 12.97_ = 19.37, *P* = 0.0002; post hoc test, 0 vs. 0.1 *P* = 0.8331, 0 vs. 0.3 *P* = 0.0065, 0 vs. 0.6 *P* = 0.0002; duration: ANOVA, *F*_2.138, 14.97_ = 0.2491, *P* = 0.7966, one-way repeated measures ANOVA with Tukey post hoc test). Session-level analysis showed that the number of nose pokes was significantly reduced during 0–15 min and 45–60 min time bins within single sessions (Fig. S3C, D). These findings suggested that blocking dopamine D_1_ and D_2_ receptors reduced the appetitive component of motivation for wheel running, whereas D_1_ receptor blockade also reduced the consummatory component, further suggesting that D_1_ and D_2_ receptor blockade exert distinct effects on appetitive behavior.

The involvement of the NAc in motivated behavior across various types of rewards has been well-documented [40–43]. Building on our previous work, which suggested that the mNAc has motivational effects on wheel running [23], we investigated the effects of suppressing neural activity in the mNAc on motivation for wheel running. Microinjection of the GABA_A_ receptor agonist muscimol (0.01 µg/side) into the mNAc significantly reduced the number of nose pokes without altering the duration of wheel running (Fig. 2E–G; nose pokes: *t*_7_ = 5.591, *P* = 0.0008; duration: *t*_4_ = 0.3356, *P* = 0.754, paired *t*-test). Subsequently, we examined the role of dopamine receptors in the mNAc in regulating motivation for wheel running. Intra-mNAc administration of SCH23390 (0.05 µg/side) significantly reduced both the number of nose pokes and duration of wheel running (Fig. 2H–J; nose pokes: *t*_9_ = 2.820, *P* = 0.0201; duration: *t*_8_ = 3.117, *P* = 0.0143, paired *t*-test). Conversely, intra-mNAc administration of raclopride (0.3 µg/side) significantly reduced the number of nose pokes without significantly altering the duration of wheel running (Fig. 2K–M; nose pokes: *t*_7_ = 4.503, *P* = 0.0028; duration: *t*_6_ = 1.186, *P* = 0.2803, paired *t*-test). These findings indicated that inhibition of neural activity and dopamine signaling via D_1_ and D_2_ receptors in the mNAc decreased the appetitive component of motivation for wheel running, whereas D_1_ receptor inhibition also reduced the consummatory component.

### Neural activity in the mNAc during appetitive and consummatory behaviors in the operant wheel running task

To investigate the temporal patterns of neural activity in the mNAc during the operant wheel running task, we expressed jGCaMP8m, a fluorescent Ca^2+^ indicator, in the mNAc, using an AAV vector and performed fiber photometry recordings during the FR10 task (Fig. 3A–C). We first confirmed that the fiber did not affect task performance in mice and that the fiber was not damaged during the wheel running task (Fig. S4). GCaMP fluorescence gradually decreased before clusters of nose pokes and transiently increased immediately after the 10th nose poke when the wheel was unlocked (Fig. 3D). To verify the neural activity associated with appetitive behavior during the wheel running task, GCaMP fluorescence was aligned to the onset of the first nose poke, assuming that motivation for wheel running would increase prior to nose-poking initiation. Representative data from one mouse revealed a gradual decrease in GCaMP fluorescence before the first nose poke (Fig. 3E; blue line: higher bound 95% extended bootstrapped CI (higher bCI) < 0, bootstrap test), and this pattern was consistently observed in the mean fluorescence across all mice (Fig. 3G). To compare fluorescence changes around the onset of the first nose poke with baseline activity, the AUC of GCaMP fluorescence was calculated from −5 to −3 s (baseline) and −1 to 1 s (around the onset of the first nose poke). The AUC around the onset of the first nose poke was significantly reduced compared to that at baseline (Fig. 3H; *t*_8_ = 4.496, *P* = 0.0020, paired *t*-test). To evaluate the effect of reward delivery on mNAc neural activity, GCaMP fluorescence was aligned to the 10th nose poke. Both representative and mean GCaMP fluorescence data showed increases following the 10th nose poke across all mice (Fig. 3F, I; blue line: higher bCI < 0, red line: lower bound 95% extended bootstrapped CI (lower bCI) > 0, bootstrap test). The AUC of GCaMP fluorescence was significantly elevated after the 10th nose poke compared to that at baseline (Fig. 3J; *t*_8_ = 6.726, *P* = 0.0001, paired *t*-test).

Next, we analyzed changes in GCaMP fluorescence during the period when the wheel was unlocked following the 10th nose poke. During this time, the mice exhibited four distinct behaviors: climbing onto the wheel, starting to run, stopping running, and leaving the wheel. Alignment of the mean GCaMP fluorescence with these behaviors revealed that the AUC of GCaMP fluorescence significantly increased when mice climbed onto the wheel and stopped running (Fig. 3K, M; blue line: higher bCI < 0, red line: lower bCI > 0, bootstrap test; climb: *t*_8_ = 2.618, *P* = 0.0307; stop: *t*_8_ = 9.520, *P* < 0.0001, paired *t*-test). However, there were no significant changes in the AUC during other behaviors or when the wheel was locked (Fig. 3L, N, O; blue line: higher bCI < 0, red line: lower bCI > 0, bootstrap test; start: *t*_8_ = 0.8628, *P* = 0.4134; leave: *t*_8_ = 2.192, *P* = 0.0598; lock: *t*_8_ = 1.056, *P* = 0.3219, paired *t*-test). Notably, almost all the events of climbing onto the wheel and starting to run occurred within the 5 s post 10th nose poke window (Fig. S5A, B, D; climb: 1.833 s, start: 1.583 s, median latency). However, the representative heatmap aligned to the 10th nose poke with annotations for these behaviors showed that the transient increase in GCaMP fluorescence occurred independently of these behaviors, suggesting that these behaviors were not related to increased neural activity observed after the 10th nose poke (Fig. S5C, D). In addition, during the wheel-unlocked period, the mean GCaMP fluorescence during wheel running was not significantly different from that during non-wheel running (Fig. S6). These findings suggested that neural activity in the mNAc decreased at the onset of appetitive behavior related to wheel running but increased when the cue signaling the availability of wheel running was presented upon the 10th nose poke.

### Dopamine release in the mNAc during appetitive and consummatory behaviors in the operant wheel running task

To investigate the changes in dopamine concentration in the mNAc, we expressed GRAB_DA2h_, a fluorescent dopamine sensor, in the mNAc using an AAV vector (Fig. 4A–C). Fiber photometry recordings were performed during the operant wheel running task to obtain real-time dopamine fluctuations associated with appetitive and consummatory behaviors. An increase in GRAB_DA2h_ fluorescence was observed before the first nose poke (Fig. 4D, E, G; blue line: higher bCI < 0, red line: lower bCI > 0, bootstrap test), with the AUC of GRAB_DA2h_ fluorescence significantly elevated compared to that at the baseline (Fig. 4H; *t*_8_ = 2.794, *P* = 0.0234, paired *t*-test). Both the representative and mean traces of GRAB_DA2h_ fluorescence across all mice revealed a transient increase following the 10th nose poke (Fig. 4D, F, I; red line: lower bCI > 0, bootstrap test). The AUC of GRAB_DA2h_ fluorescence after the 10th nose poke was significantly greater than that before the 10th nose poke (Fig. 4J; *t*_8_ = 5.554, *P* = 0.0005, paired *t*-test).

During the wheel-unlocked period following the 10th nose poke, there were no significant changes in the AUC of GRAB_DA2h_ fluorescence before any of the aforementioned four behaviors compared to that at the baseline (Fig. 4K–N; blue line: higher bCI < 0, red line: lower bCI > 0, bootstrap test; climb: *t*_8_ = 1.180, *P* = 0.2718; start: *t*_8_ = 2.267, *P* = 0.0532; stop: *t*_8_ = 0.6255, *P* = 0.5490; leave: *t*_8_ = 1.456, *P* = 0.1836, paired *t*-test). However, the AUC of GRAB_DA2h_ fluorescence significantly decreased following the onset of wheel locking (Fig. 4O; blue line: higher bCI < 0; lock: *t*_8_ = 3.941, *P* = 0.0043, paired *t*-test). These results suggested that dopamine release in the mNAc increased before the onset of appetitive behavior and in response to cues signaling the availability of wheel running. Conversely, dopamine release decreased following the perception of reward unavailability.

### The role of dopamine receptors in modulating neural activity changes in the mNAc during appetitive and consummatory behaviors in the operant wheel running task

To elucidate the relationship between temporal changes in neural activity and dopamine release, we measured neural activity in the mNAc during the operant wheel running task following systemic administration of dopamine receptor antagonists. Fiber photometry recordings revealed that systemic administration of SCH23390 (0.05 mg/kg) attenuated the decrease in GCaMP fluorescence around the onset of the first nose poke compared to that in vehicle-treated mice (Fig. 5A; gray line: vehicle higher bCI < 0, magenta line: SCH23390 higher bCI < 0, bootstrap test; orange line: vehicle vs SCH23390 *P* < 0.05, permutation test). The AUC of GCaMP fluorescence during this period was significantly reduced in SCH23390-treated mice compared to that in the controls (Fig. 5A; *t*_5_ = 2.996, *P* = 0.032, paired *t*-test). In contrast, both vehicle- and SCH23390-treated mice exhibited significant increases in GCaMP fluorescence following the 10th nose poke, although the magnitude of this increase was slightly, but not significantly, lower in SCH23390-treated mice (Fig. 5B; gray line: vehicle lower bCI > 0 or higher bCI < 0, magenta line: SCH23390 lower bCI > 0 or higher bCI < 0, bootstrap test; *t*_5_ = 2.458, *P* = 0.0574, paired *t*-test). In contrast, raclopride (0.3 mg/kg) treatment did not significantly affect changes in the AUC of GCaMP fluorescence around the onset of the first nose poke or the magnitude of increase after the 10th nose poke (Fig. 5C, D; gray line: vehicle lower bCI > 0 or higher bCI < 0, cyan line: raclopride lower bCI > 0 or higher bCI < 0, bootstrap test; orange line: vehicle vs raclopride *P* < 0.05, permutation test; first: *t*_5_ = 2.092, *P* = 0.0906; 10th: *t*_5_ = 1.986, *P* = 0.1038, paired *t*-test).

Session-level analysis revealed that D_1_ receptor blockade consistently reduced the AUC of GCaMP fluorescence around the onset of the first nose poke, whereas D_2_ receptor blockade consistently increased the response after the 10th nose poke, although no significant time-dependent changes were observed within single sessions (Fig. S7). Additionally, during the wheel-unlocked period, neither SCH23390 nor raclopride significantly affected the AUC of GCaMP fluorescence associated with the four distinct behaviors or after the wheel was locked (Fig. S8). Furthermore, the mean GCaMP fluorescence during wheel running was not significantly altered by either SCH23390 or raclopride (Fig. S9).

These findings suggested that dopamine signaling through D_1_ receptors played a significant role in reducing neural activity in the mNAc around the onset of appetitive behavior during operant wheel running task.

## Discussion

In this study, we explored how neural activity and dopamine signaling in the mNAc influence motivated behavior in wheel running using an operant conditioning task distinguishing the appetitive and consummatory phases. Following previous reports [27, 28], appetitive and consummatory behaviors were assessed by nose poke number and wheel running duration, respectively. The NAc regulates both phases [44–46], primarily modulating the appetitive phase via dopamine [27, 47]. We found that mNAc inactivation by muscimol reduced appetitive but not consummatory behavior, corroborating earlier findings on the NAc’s role in wheel running motivation [20, 23]. Furthermore, based on the photometry data shown in Fig. 3, the increased c-Fos expression following voluntary wheel running observed in the previous studies [20, 23] may partly reflect transient neural activation associated with perceiving the running wheel, initiating climbing onto it, or terminating running. Supporting this possibility, our muscimol experiment showed that inhibition of mNAc neural activity did not affect running duration, suggesting that the neural activation observed in both the present study and previous c-Fos studies [20, 23] may not be related to the consummatory component of motivated behavior. To test this hypothesis, neural inhibition methods with higher temporal precision, such as optogenetics, will be needed. Conversely, NAc inactivation enhanced food-reinforced operant responses and food intake [44, 48, 49], suggesting distinct reward processing mechanisms for food versus wheel running.

### The role of dopamine receptors in the mNAc in appetitive and consummatory behavior in the operant wheel running task

Systemic D_1_ or D_2_ receptor blockade reduced operant responding for wheel running, highlighting the role of dopamine signaling through these receptors in modulating appetitive behavior. Similar effects were observed when D_1_ and D_2_ receptor antagonists were administered directly into the mNAc. These results are consistent with those of previous studies demonstrating that systemic or intra-NAc administration of D_1_ or D_2_ receptor antagonists suppresses appetitive behavior in operant tasks reinforced by food or addictive drugs [50–56]. Collectively, these findings underscored the critical role of dopamine signaling via D_1_ and D_2_ receptors in the NAc in regulating appetitive behavior, not only for food and addictive drugs but also for wheel running. Furthermore, our data indicated that both systemic and intra-mNAc D_1_ receptor blockade reduced the duration of wheel running, suggesting that D_1_ signaling in the mNAc contributes to consummatory behavior. However, previous studies have reported minimal effects of D_1_ receptor signaling on the consummatory behavior of food rewards [57, 58]. This apparent discrepancy may likely arise from differences in reward types. It has been suggested that distinct neural processing mechanisms exist for natural rewards and addictive drugs [59–62] and even among different types of natural rewards [63–65]. For instance, Fernandes et al. [7] demonstrated that leptin suppresses CPP for wheel running by inhibiting VTA dopaminergic neurons, supporting the importance of dopaminergic signaling in the rewarding effects of wheel running and further corroborating our hypothesis. In addition, several lines of evidence show that pharmacological dopamine depletion and D_2_ receptor blockade shift choice behavior from the wheel-running reward to the food reward in the T-maze task, further supporting our hypothesis [66–70]. Alternatively, the reduction in wheel running duration observed in our study may reflect decreased locomotor activity, as dopamine receptors are known to modulate motor function [71, 72]. However, previous studies have demonstrated that even higher doses of SCH23390 than those used in this study did not reduce locomotor activity in mice during open field tests [73–75]. Therefore, it is more likely that the observed reduction in wheel running following D_1_ receptor blockade reflected decreased motivated behavior for wheel running rather than a general suppression of locomotor activity.

Session-level analyses suggest that systemic D_1_ and D_2_ receptor blockade affects appetitive behavior in different within-session dynamics. In contrast to the previous report [76], we did not observe any apparent extinction-like effects on appetitive behavior by dopamine receptor inhibition. This discrepancy may be due to differences in the pharmacological profiles of the antagonists used, reward type, and response rate.

### Neural activity and dopamine release in the mNAc during appetitive behavior in the operant wheel running task

We observed that the mNAc neural activity decreased before appetitive behavior onset for wheel running and increased following reward-available cue presentation, corroborating earlier findings from food- and cocaine-reinforced operant tasks [77–79]. This decrease, which is undetectable by c-Fos analysis, may signify appetitive behavior initiation, whereas the post-cue increase possibly denotes the reward value that reinforces subsequent reward-seeking behavior [78, 80]. This decrease in neural activity during appetitive behavior appears to be inconsistent with the finding that intra-mNAc muscimol administration suppresses appetitive behavior. However, considering the effects of the D_1_ receptor antagonist on neural activity and behavior, it is possible that muscimol disrupts one or both of these transient neural activity patterns through sustained inhibition, thereby impairing appetitive behavior. To further clarify this relationship, precise optogenetic studies are required to identify which change directly affects appetitive behavior.

Cyclic voltammetry studies have previously demonstrated that dopamine concentrations in the NAc increase both before lever presses for food and cocaine [81–83] and after the presentation of a reward-predictive cue, suggesting the importance of dopamine signaling in appetitive behavior [84, 85]. Consistent with these findings, we observed that dopamine release in the mNAc increased before the onset of appetitive behavior and after the presentation of the reward-available cues in the operant wheel running task. Given that increases in NAc dopamine levels are known to promote food- and cocaine-seeking behaviors [81, 86], these transient surges in mNAc dopamine likely play a modulatory role in appetitive component of motivated wheel running behavior.

We revealed that dopamine D_1_ receptor blockade attenuated the decrease in mNAc neural activity observed around the onset of appetitive behavior associated with wheel running. The majority (>95%) of neurons in the NAc are medium spiny neurons (MSNs), the activity of which is strongly regulated by excitatory glutamatergic inputs and modulated by dopaminergic signaling [87–90]. Several in vitro electrophysiological studies demonstrated that dopamine attenuates glutamatergic synaptic transmission in the NAc through presynaptic D_1_ receptor activation [91–94]. Additionally, recent evidence suggests that dopamine reduces excitatory synaptic transmission through D_1_ receptor signaling in astrocytes in the NAc [95]. Thus, dopamine may attenuate mNAc neural activity by suppressing excitatory inputs to the mNAc, thereby contributing to an increase in appetitive behavior.

D_1_ receptor blockade suppressed the increase in mNAc neural activity following the reward-available cue presentation. The NAc receives excitatory glutamatergic inputs from regions including the medial prefrontal cortex, ventral hippocampus, basolateral amygdala, and paraventricular thalamus, which are involved in motivated behavior [96–100]. The rise in neural activity following cue presentation may stem from heightened glutamatergic transmissions from these regions. Further research is necessary to identify the specific glutamatergic inputs to the mNAc essential for motivated wheel running behavior.

Notably, D_2_ receptor blockade did not alter overall mNAc neural activity but significantly reduced appetitive behavior when administered systemically or into the mNAc. D_2_ receptors in the NAc are found on MSNs and cholinergic interneurons (ChIs) [89, 101]. MSN activity is suppressed by D_2_ receptor stimulation via the Gi/o signaling pathway [102], whereas ChIs inhibit MSNs through both nicotinic receptor activation and muscarinic receptor-mediated inhibition of glutamatergic inputs [103–106]. Stimulation of D_2_ receptors on ChIs likely inhibits these interneurons via the same signaling pathway, disinhibiting MSNs and enhancing their activity. Therefore, the net effects of D_2_ receptor antagonism on mNAc neural activity, as observed by fiber photometry, may be negligible due to the balancing of MSN activity. Further studies should include recordings from specific neural subpopulations to elucidate the effect of D_2_ receptor blockade on neural activity in the mNAc.

### Neural activity and dopamine release in the mNAc during consummatory behavior in the operant wheel running task

During the consummatory phase of the wheel running task, changes in neural activity and dopamine release in the mNAc mirrored findings from those reported under food-reward paradigms [107, 108]. Specifically, mNAc neural activity increased when mice climbed onto the wheel, highlighting the role of mNAc neural activity in approaching wheel running rewards. This observation is consistent with previous studies showing that NAc firing increases when rats enter a food reward receptacle [107]. Additionally, we found that dopamine levels in the mNAc transiently decreased after the end of the wheel running period. This result aligns with prior reports of decreased dopamine levels in the ventral striatum in response to cues signaling the unavailability of sucrose rewards [108]. Although burst firing of VTA dopaminergic neurons has been reported at both the onset and offset of wheel running [25], dopamine release remained unchanged during the actual wheel running behavior in our study. It is important to note that dopamine release in the NAc does not always directly correspond to dopaminergic neural activity [83].

Neural activity in the mNAc during the wheel running period was not altered by D_1_ receptor blockade, despite the reduction in consummatory behavior following systemic and intra-mNAc D_1_ receptor antagonist administration. A previous study has reported that even among D_1_-positive MSNs, there are distinct subpopulations that exhibit different activity patterns in response to stimuli [109]. Therefore, it is possible that our fiber photometry recordings could not detect subpopulation-specific changes. Nonetheless, D_1_ receptor antagonism may modulate the activity of specific neural subpopulations in the mNAc, and such undetected changes could underlie the observed alterations in consummatory behavior. Further insight into the effects of D_1_ receptor antagonists on consummatory behavior would be gained by single cell calcium imaging.

A limitation of this study is that only male mice were examined. Previous studies have reported sex differences in wheel running activity and in the acquisition of operant conditioning [110, 111]. Thus, future research should investigate potential sex differences in motivated behavior for wheel running.

In conclusion, the operant wheel running task showed that neural activity and dopamine signaling through D_1_ and D_2_ receptors in the mNAc are important for motivated behavior. Specifically, dopamine release and the subsequent neural activity changes mediated by D_1_ receptors in the mNAc are pivotal for this behavior. Further investigation into the neural mechanisms underlying these activity changes, along with the identification of neuronal subtypes within the mNAc, may provide greater insight into the neural basis of mental disorders characterized by aberrant motivation for natural rewards, such as major depressive disorder and behavioral addictions.

## Supplementary information

Supplemental methods & Figs for readers

## Data Availability

Data will be available upon request to Dr. Katsuyuki Kaneda (k-kaneda@p.kanazawa-u.ac.jp).
