# Supplementary material for: Medial nucleus accumbens dopamine receptors modulate motivation for wheel running in male mice
Source: Neuropsychopharmacology. 2025 Jun 12;50(13):1982–92. doi: 10.1038/s41386-025-02136-w (PMC12603203; doi:10.1038/s41386-025-02136-w)
Supplement: Supplementary file 1 — Supplemental methods & Figs for readers [file 41386_2025_2136_MOESM1_ESM.pdf]

Supplementary Materials and Methods

Adeno-associated virus (AAV) vector production

AAVs were prepared according to a previously described protocol with slight modifications [1–3]. Lenti-X 293T cells (Clontech, Palo Alto, CA, USA) were grown to 60–70% confluency, and 2.7 μg of pHelper, 1.7 μg of pAAV-DJ Rep-Cap (Cell biolabs, San Diego, CA, USA), and 1.5 μg of pGP-AAV-syn-jGCaMP8m-WPRE (Addgene plasmid #162375)[4] or pAAV-hSyn-GRAB$_{DA2h}$ (Addgene plasmid #140554) [5] were transfected with polyethylenimine (Polysciences, Warrington, PA, USA). After 60–72 h of incubation, the supernatant and cells were collected. The cell suspension was frozen at −80 °C for 10 min, incubated at 34 °C for 2 min, and then vortexed for 1 min. This freeze-thaw cycle was repeated three more times. After 0.5 μL of benzonase nuclease (Sigma, St Louis, MO, USA) was added, the lysate was incubated at 37 °C for 30 min, followed by centrifugation at 18,000 g for 10 min. The supernatant was then transferred to a new centrifuge tube, and this centrifuge step was repeated three more times. This lysate was aliquoted and stored at −80 °C until use.

Operant wheel running task

Behavioral setup. Experiments were conducted in a custom-built operant chamber (length, 21.8 cm; width, 16.5 cm; height, 20 cm). The lateral wall of the chamber had two nose poke ports, each consisting of a photo interrupter, spaced 14 cm apart, and a running wheel (21 cm in diameter) positioned between the two ports. Two yellow cue lights and one white cue light were located above the nose poke ports

and the wheel, respectively. The wheel was locked by a brake pad and unlocked in accordance with experimental conditions. A yellow cue light above the active nose poke port and a white cue light were turned on when the mice completed the operant requirement. The yellow light turned off after 10 seconds, and the white light turned off when the wheel was locked. Revolutions of the wheel were counted every 1 s by a rotary encoder (RES20D-50-201-1; Nidec, Kyoto, Japan) attached to the wheel. The onset of wheel rotation was defined as a rotation of > 25° within 1 s. If the wheel did not rotate > 25° within 1 s for 2 s, we defined the rotation stop. A custom program running on an Arduino Uno and an Arduino Nano microcontroller (Arduino, Ivrea, Italy) was used to control the experimental logic.

Training. First, mice were habituated to the chamber and allowed to run freely on the wheel for 60 min per day for at least 3 days until they ran > 400 m and for > 20 min. Second, mice were trained on the fixed ratio (FR) 1 schedule (FR1), in which the wheel was unlocked for 1 min immediately after each nose poke to the active nose poke port. Once they met the following criteria: (1) the number of wheel release in a session deviated by ≤ 20% across three consecutive sessions, and (2) the percentage of inactive nose pokes [%inative = (inactive nose pokes / (inactive nose pokes + active nose pokes))×100] was < 20%, they moved on to the FR3, then the FR5, and finally FR10 task after satisfying the same criteria in each schedule. Systemic drug injections, intra-mNAc drug infusions, and fiber photometry recordings were conducted in the FR10 task after the mice had met these criteria. For systemic administrations, a dopamine D_1-like receptor antagonist SCH23390 (0.025, 0.05, and 0.1 mg/kg) and a dopamine D_2-like receptor antagonist raclopride (0.1, 0.3, and 0.6 mg/kg) were injected

intraperitoneally 15 min and 5 min before testing, respectively, according to a Latin-square design. For local administrations, 33-gauge stainless-steel infusion cannulae were inserted bilaterally through the guide cannulae, extending 0.5 mm beyond the guide tip to reach the medial NAc (mNAc). A $GABA_A$ receptor agonist muscimol (0.01 μg/side), SCH23390 (0.05 μg/side), raclopride (0.3 μg/side), or saline was administered in a 0.2-μL volume at a rate of 0.2 μL/min. Each drug and saline were administered in a counterbalanced manner. In fiber photometry experiments, SCH23390 (0.05 mg /kg), raclopride (0.3 mg/kg), or vehicle was administered in a counterbalanced manner. The systemic doses of SCH23390 and raclopride were selected based on previous reports [6,7], respectively. The intra-mNAc doses of SCH23390 and raclopride were selected based on a previous report [8]. Following a previous study [9], data from mice that did not successfully unlock the wheel (i.e., failed to obtain the wheel running reward) were excluded from the analysis of running duration.

Stereotaxic surgeries

Stereotaxic surgeries were conducted using a stereotaxic frame (Narishige, Tokyo, Japan) according to the Brain Atlas [10]. Mice were anesthetized with a combination of medetomidine (0.3 mg/kg, i.p.; Meiji Seika Pharma, Tokyo, Japan), midazolam (4 mg/kg, i.p.; Sandoz, Tokyo, Japan) and butorphanol (5 mg/kg, i.p.; Meiji Seika Pharma). For local administration experiments, each mouse that met the criteria in the FR10 task was bilaterally implanted with a 25-gauge stainless-steel guide cannula (o.d., 0.51 mm; i.d., 0.26 mm) above the mNAc (AP +1.4 mm, ML ±0.8 mm, DV −4.2 mm to the bregma, at

a 19° angle from the vertical axis in the mediolateral plane). After surgery, mice were housed to recover for at least 3 days, and then retrained on the FR10 task until they again met the criteria described above. For fiber photometry experiments, 500 nL of AAVdj-hSyn-$GRAB_{DA2h}$ (2.3×10^{10} vg/mL) or AAVdj-hSyn-jGCaMP8m (4.5×10^{9} vg/mL) was unilaterally injected into the mNAc (AP +1.4 mm, ML 0.8 mm, DV −5.0 mm to the bregma) and a fiber optic cannula (400 µm core, 0.50 NA; RWD, Shenzhen, China) was implanted into the same site. Following a 2-week recovery period, operant wheel running training was initiated.

Fiber photometry

A basic fiber photometry system (FPS_1S_GCaMP; Doric Lenses, Quebec, Canada) was used. A sinusoidal 465-nm LED (~30 µW, 572.205-Hz; CLED465; Doric Lenses) and a sinusoidal isosbestic 405-nm LED (driven with the same current amplitude as the 465-nm LED, 208.616-Hz; CLED405; Doric Lenses) were coupled to a filter cube (iFMC4-G2_IE(400-410)_E(460-490)_F(500-550)_S; Doric Lenses) and delivered to the mNAc via a fiber patch cord connected to the fiber optic cannula. Fluorescence signals were transmitted through the same patch cord to a photodetector (iFMC4-G2_IE(400-410)_E(460-490)_F(500-550)_S; Doric Lenses). Raw photometry signals were collected at 12 kHz and de-interleaved to separate reference (405 nm) and experimental (465 nm) signals using Doric Neuroscience Studio software (Doric Lenses). Python scripts [11] were employed for the analysis of photometry signals after decimation by a factor of 1000. First, each signal underwent artifact removal

using a moving average technique and adaptive iteratively reweighted Penalized Least Squares for photobleaching correction. Next, both the reference and experimental signals were normalized to z-scores, calculated as $F_n - F_{median}/F_{SD}$, where F_n is the signal at time n, and F_{median} and F_{SD} are the median and standard deviation of the total signal during the operant wheel running task for each channel, respectively. The z-scored reference signal was then regressed on and subtracted from the z-scored experimental signal to obtain zdF/F_0 (z-scored delta $F_{experimental}$ over $F_{reference}$). To quantify the effect of wheel running on mNAc neural activity, the mean zdF/F_0 was calculated separately during periods when the mice were running on the wheel and when they were not, using a time window from 10 to 60 s after the 10th nose poke. This time window was chosen to avoid contamination from the transient increase in neural activity typically observed immediately after the 10th nose poke. For systemic administrations, the dopamine D_1-like receptor antagonist SCH23390 (0.05 mg/kg) and the dopamine D_2-like receptor antagonist raclopride (0.3 mg/kg) were injected intraperitoneally 15 min and 5 min before testing, respectively. Each drug and saline were administered in a counterbalanced manner.

Histology

To confirm infusion sites in the mNAc, mice were decapitated, and brains were rapidly removed and frozen in powdered dry ice. Coronal sections (50 μm) were prepared on a cryostat (Leica CM3050S; Leica Biosystems, Nussloch, Germany), mounted onto slides, stained with thionin, and examined under a brightfield microscope (BZ-X810, Keyence, Osaka, Japan). To confirm viral expression and fiber

optic cannula placements, mice were deeply anesthetized with chloral hydrate (400 mg/kg, i.p.) and transcardially perfused with PBS followed by 4% paraformaldehyde (PFA, Nacalai Tesque, Kyoto, Japan) in PBS. After perfusion, brains were removed, equilibrated in 20% sucrose in PBS overnight, and frozen using dry ice. Coronal sections (30 μm-thick) were prepared using a cryostat and stored at −80 °C until immunohistochemical processing. For immunohistochemistry, the sections were permeabilized in PBS containing 0.3% Triton-X 100 and incubated overnight at room temperature with a rabbit polyclonal anti-green fluorescent protein (GFP) antibody (1:3000; 598, MBL life science, Tokyo, Japan), followed by incubation with Alexa Fluor 488-labeled donkey anti-rabbit IgG (1:500; ab150043, Abcam, Cambridge, UK) for 2 h at room temperature. After washing in PBS, the sections were mounted on glass with Fluoromount-G (SouthernBiotech, Birmingham, AL, USA). Immunofluorescence was visualized using a microscope (BZ-X810).

Statistical Analyses

Data are expressed as means ± SEM. Statistical tests were performed using Student's *t*-test, paired *t*-test, one-way repeated-measures analysis of variance (ANOVA) with the Tukey *post hoc* test, two-way repeated-measures ANOVA with the Dunnett's *post hoc* test in GraphPad Prism 9 software (GraphPad Software, La Jolla, CA, USA). In the presence of missing values due to no nose pokes or no reward obtained within 15 min (Fig. S3B, D and S6), data were analyzed using a mixed-effects model fitted with Restricted Maximum Likelihood (REML) estimation instead of two-way repeated-measures

ANOVA using GraphPad Prism 9. Differences with $P < 0.05$ were considered statistically significant. In the fiber photometry experiments, bootstrap and permutation tests were used to assess the differences in fluorescent signals from baseline and control groups, respectively, using a homemade python script using Scypi [12]. For the bootstrap test, 1,000 bootstrapped means were obtained by random resampling with replacement. Then, 95% bootstrapped confidence interval (bCI) was calculated and expanded by a factor of $\sqrt{n(n-1)}$ to counter small sample narrowness bias. For the permutation test, 1,000 random permutations were used to obtain P values. Periods where the expanded bCIs did not contain the 0 (bootstrapped test) for at least 0.5 s, or where $P < 0.05$ (permutation test) for at least 0.5 s, were considered statistically significant. Sample sizes were determined based on related reports [9,13,14] and were not predetermined by calculation.

References

1. Kawai H, Bouchekioua Y, Nishitani N, Niitani K, Izumi S, Morishita H, et al. Median raphe serotonergic neurons projecting to the interpeduncular nucleus control preference and aversion. Nat Commun. 2022;13:7708.
2. Nagai Y, Kisaka Y, Nomura K, Nishitani N, Andoh C, Koda M, et al. Dorsal raphe serotonergic neurons preferentially reactivate dorsal dentate gyrus cell ensembles associated with positive experience. Cell Rep. 2023;42:112149.
3. Saito A, Murata H, Niitani K, Nagasaki J, Otoda A, Chujo Y, et al. Social defeat stress enhances

the rewarding effects of cocaine through α1A adrenoceptors in the medial prefrontal cortex of mice. Neuropharmacology. 2024;242:109757.

4. Zhang Y, Rózsa M, Liang Y, Bushey D, Wei Z, Zheng J, et al. Fast and sensitive GCaMP calcium indicators for imaging neural populations. Nature. 2023;615:884–891.

5. Sun F, Zhou J, Dai B, Qian T, Zeng J, Li X, et al. Next-generation GRAB sensors for monitoring dopaminergic activity in vivo. Nat Methods. 2020;17:1156–1166.

6. Caine SB, Thomsen M, Gabriel KI, Berkowitz JS, Gold LH, Koob GF, et al. Lack of self-administration of cocaine in dopamine D1 receptor knock-out mice. J Neurosci. 2007;27:13140–13150.

7. Heath CJ, Bussey TJ, Saksida LM. Motivational assessment of mice using the touchscreen operant testing system: effects of dopaminergic drugs. Psychopharmacology (Berl). 2015;232:4043–4057.

8. Young EA, Dreumont SE, Cunningham CL. Role of nucleus accumbens dopamine receptor subtypes in the learning and expression of alcohol-seeking behavior. Neurobiol Learn Mem. 2014;108:28–37.

9. Muguruza C, Redon B, Fois GR, Hurel I, Scocard A, Nguyen C, et al. The motivation for exercise over palatable food is dictated by cannabinoid type-1 receptors. JCI Insight. 2019;4.

10. Franklin KBJ, Paxinos G. The mouse brain in stereotaxic coordinates 3rd ed. Academic Press: San Diego, CA; 2007.

11. Martianova E, Aronson S, Proulx CD. Multi-Fiber Photometry to Record Neural Activity in Freely-Moving Animals. J Vis Exp. 2019;2019.

12. Jean-Richard-Dit-Bressel P, Clifford CWG, McNally GP. Analyzing Event-Related Transients: Confidence Intervals, Permutation Tests, and Consecutive Thresholds. Front Mol Neurosci. 2020;13:14.

13. Muir J, Lorsch ZS, Ramakrishnan C, Deisseroth K, Nestler EJ, Calipari ES, et al. In Vivo Fiber Photometry Reveals Signature of Future Stress Susceptibility in Nucleus Accumbens. Neuropsychopharmacology. 2018;43:255–263.

14. Mazzone CM, Liang-Guallpa J, Li C, Wolcott NS, Boone MH, Southern M, et al. High-fat food biases hypothalamic and mesolimbic expression of consummatory drives. Nat Neurosci. 2020;23:1253–1266.

Supplementary Figures

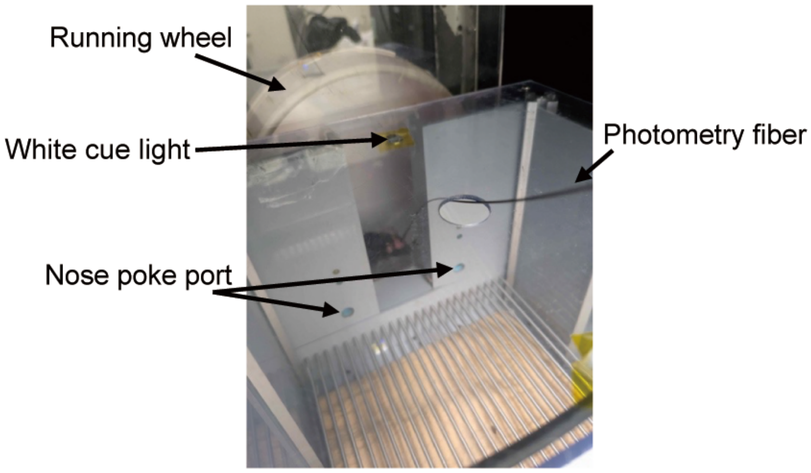

Fig. S1. Behavioral setup with photometry fiber.

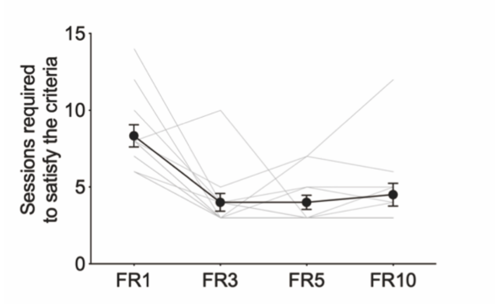

Fig. S2. The number of sessions required to meet the criteria. (n = 12). Data are expressed as means ± SEM.

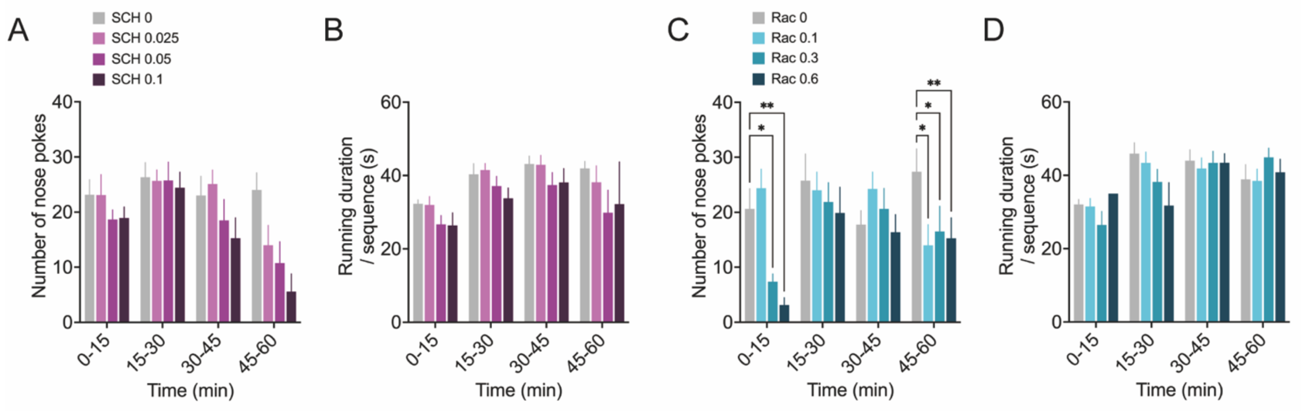

Fig. S3. Effect of systemic dopamine D_1 and D_2 receptor inhibition on motivated behavior for wheel running within single sessions. **A, B** Effects of the D_1 receptor antagonist SCH23390 on the number of nose pokes (**A**) and running duration (**B**) every 15 min during sessions (n = 12; nose poke: interaction $F_{4.050, 44.55} = 2.180$, $P = 0.0859$; time $F_{2.368, 26.05} = 12.54$, $P < 0.0001$; drug $F_{2.183, 24.01} = 5.554$, $P = 0.0089$, two-way repeated measures ANOVA; running duration: interaction $F_{3.571, 28.57} = 0.5320$, $P = 0.6940$; time $F_{1.593, 17.52} = 12.60$, $P = 0.0008$; drug $F_{1.258, 13.83} = 7.157$, $P = 0.0140$, mixed-effects model). **C, D** Effects of the D_2 receptor antagonist raclopride on the number of nose pokes (**C**) and running duration (**D**) every 15 min during sessions (n = 8; nose poke: ANOVA, interaction $F_{3.445, 24.12} = 3.053$, $P = 0.0420$; time $F_{2.184, 15.29} = 3.560$, $P = 0.0505$; drug $F_{1.837, 12.86} = 18.41$, $P = 0.0002$; *post hoc* test, 0-15: 0 vs.0.3 *$P = 0.0379$, 0 vs. 0.6 **$P = 0.0046$, 45-60: 0 vs. 0.1 *$P = 0.0329$, 0 vs.0.3 *$P = 0.0161$, 0 vs. 0.6 **$P = 0.0034$, two-way repeated measures ANOVA with Dunnett's *post hoc* test; running duration: interaction $F_{9, 42} = 1.890$, $P = 0.0801$; time $F_{3, 21} = 5.815$, $P = 0.0047$; drug $F_{3, 21} = 0.4097$, $P = 0.7477$, mixed-effects model).

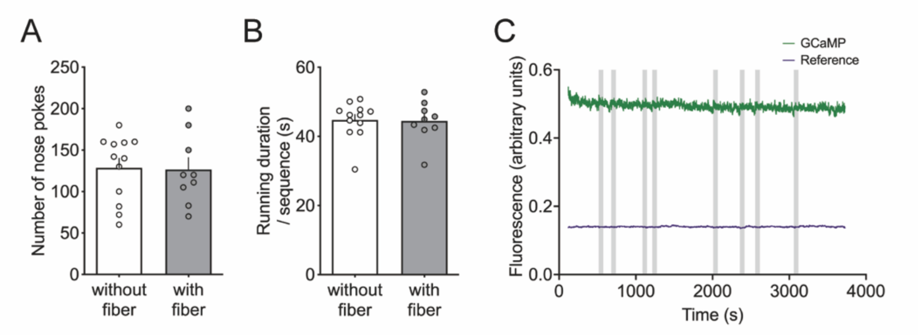

Fig. S4. Effects of fiber tethering on mouse behaviors and of wheel running on photometry recordings. **A, B** The number of nose pokes (**A**) and the duration of wheel running (**B**) in the last session that met the training criteria, with or without fiber tethering (n = 9–12; nose pokes: $t_{19} = 0.1070$, $P = 0.9159$; duration: $t_{19} = 0.1190$, $P = 0.9065$, Student's *t*-test). The data of the without fiber group are the same as in Fig. 1. **C** Representative traces of raw GCaMP (green) and reference (purple) signals recorded throughout the FR10 session. Gray shadings indicate periods when the wheel was unlocked. Data are expressed as means ± SEM.

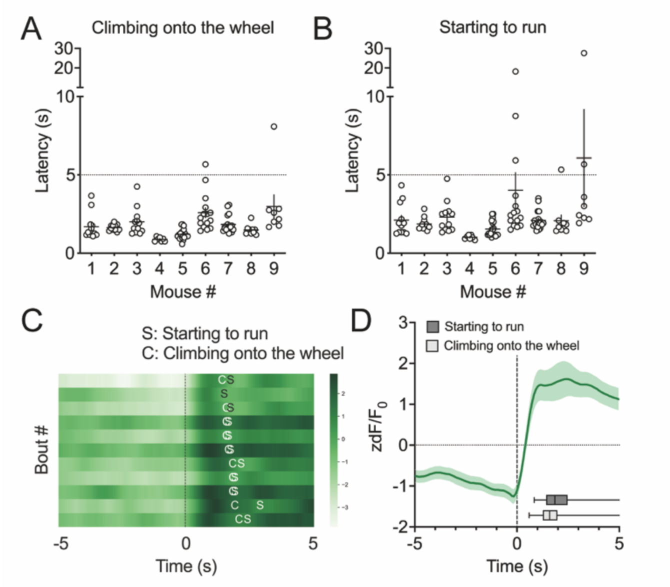

Fig. S5. Effect of climbing onto the wheel and starting to run on the transient increase in mNAc neural activity after the tenth nose pokes in the FR10 task. **A, B** The latency from the tenth nose pokes to the onset of climbing onto the wheel (**A**) and starting to run (**B**). **C** The representative heatmap aligned to the tenth nose pokes, with annotations for the onset of climbing onto the wheel (“C”) and starting to run (“S”). **D** Mean GCaMP signals around the tenth nose pokes (n = 9; same as in Fig. 3I, bottom) with box-and-whisker plots of the onset of climbing onto the wheel and starting to run.

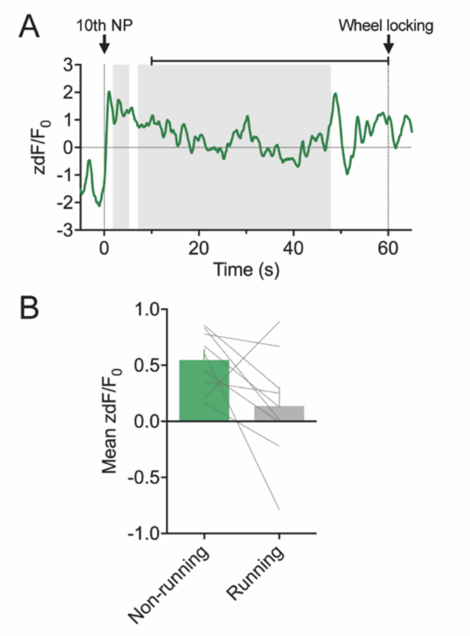

Fig. S6. Effect of wheel running on mNAc neural activity. **A** Representative GCaMP signals during the wheel-unlocked period following the 10th nose poke. Gray shadings indicate periods when the mouse was running on the wheel. Horizontal black line indicates the time window used for analysis. **B** Mean GCaMP signals during periods when the mice were running on the wheel (Running) and when they were not running on the wheel (Non-running) (n = 9; $t_8 = 2.173$, $P = 0.0616$, paired *t*-test).

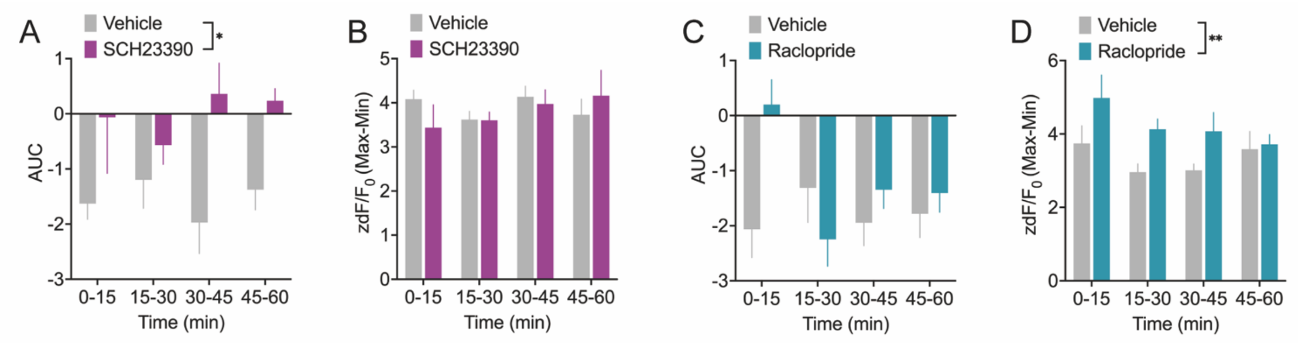

Fig. S7. Effect of systemic dopamine D_1 and D_2 receptor inhibition on changes in mNAc neural activity during appetitive behavior for wheel running within single sessions. **A, B** AUC of mean GCaMP signals from −1 s to 1 s aligned with the first nose pokes (**A**) and increase rate of mean GCaMP signals from minimum to maximum aligned with the tenth nose pokes (**B**) in the FR10 schedule every 15 min during sessions in vehicle- and SCH23390 (0.05 mg/ kg)-treated mice (n = 6; AUC: interaction $F_{1.310, 2.619}$ = 0.9229, P = 0.4501; time $F_{1.846, 9.232}$ = 0.07957, P = 0.9119; drug $F_{1, 5}$ = 13.85, *P = 0.0137; Max-Min: interaction $F_{0.8617, 1.436}$ = 1.774, P = 0.3418; time $F_{1.580, 7.898}$ = 1.101, P = 0.3618; drug $F_{1, 5}$= 1.281, P = 0.3090, mixed-effects model). **C, D** AUC of mean GCaMP signals from −1 s to 1 s aligned with the first nose pokes (**C**) and increase rate of mean GCaMP signals from minimum to maximum aligned with the tenth nose pokes (**D**) in the FR10 schedule every 15 min during sessions in vehicle- and raclopride (0.3 mg/kg)-treated mice (n = 6; AUC: interaction $F_{1.079, 4.315}$ = 6.711, P = 0.0553; time $F_{1.394, 6.970}$ = 1.836, P = 0.2250; drug $F_{1, 5}$ = 2.974, P = 0.1452; Max-Min: interaction $F_{1.512, 5.040}$ = 1.730, P = 0.2604; time $F_{1.956, 9.778}$ = 3.308, P = 0.0806; drug $F_{1, 5}$= 19.23, **P = 0.0071, mixed-effects model).

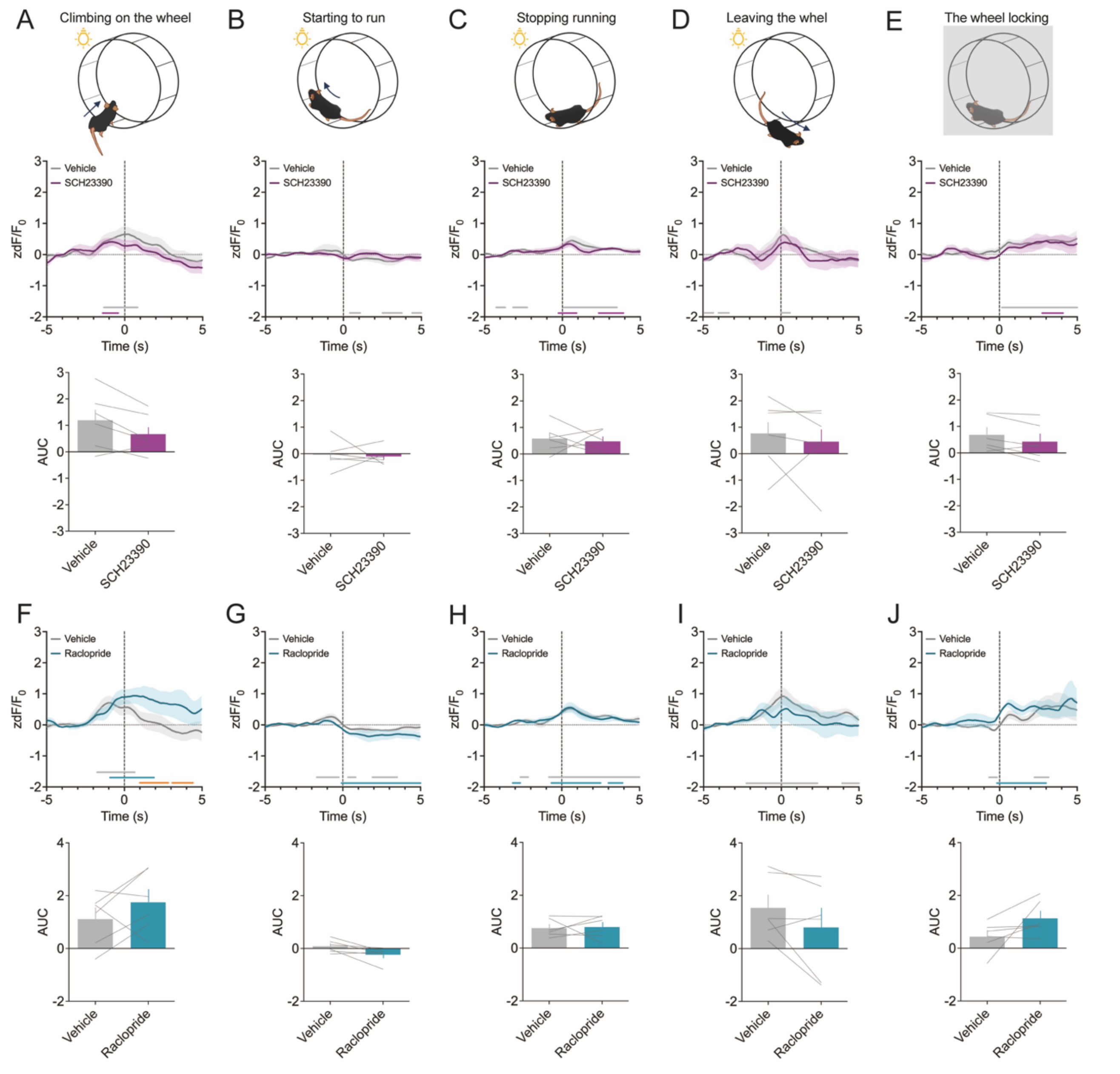

Fig. S8. Effect of systemic administration of dopamine antagonists on neural activity in the mNAc during the wheel-unlocked period. **A–E** Mean GCaMP signals and AUC of mean GCaMP signals from −5 s to −3 s and −1 s to 1 s aligned with the onset of climbing on the wheel (**A**), starting to run on the wheel (**B**), stopping running on the wheel (**C**), leaving the wheel (**D**) and AUC of mean GCaMP signals from −2 s to 0 s and 0 s to 2 s aligned with the onset of the wheel locking (**E**) in vehicle- and SCH23390-treated mice (n = 6; climb: $t_5 = 2.422$, $P = 0.0600$; start: $t_5 = 0.2321$, $P = 0.8257$; stop: $t_5 = 0.3324$, $P =$

0.7531; leave: $t_5 = 0.5860$, $P = 0.5833$; lock: $t_5 = 2.166$, $P = 0.0825$, paired *t*-test). Vertical dashed lines indicate the onset of the event, and horizontal dashed lines indicate the baseline. Gray and magenta lines indicate statistically significant differences from 0 (bootstrap test, higher bound 95% extended bootstrapped CI < 0 or lower bound 95% extended bootstrapped CI > 0; gray: vehicle, magenta: SCH23390). **F–J** Mean GCaMP signals and AUC of mean GCaMP signals from −5 s to −3 s and −1 s to 1 s aligned with the onset of climbing on the wheel (**F**), starting to run on the wheel (**G**), stopping running on the wheel (**H**), leaving the wheel (**I**) and AUC of mean GCaMP signals from −2 s to 0 s and 0 s to 2 s aligned with the onset of the wheel locking (**J**) in vehicle- and raclopride-treated mice (n = 6; climb: $t_5 = 1.283$, $P = 0.2556$; start: $t_5 = 2.515$, $P = 0.0535$; stop: $t_5 = 0.1790$, $P = 0.8649$; leave: $t_5 = 1.638$, $P = 0.1624$; lock: $t_5 = 1.896$, $P = 0.1165$, paired *t*-test). Vertical dashed lines indicate the onset of the event, and horizontal dashed lines indicate the baseline. Gray and cyan lines indicate statistically significant differences from 0 (bootstrap test, higher bound 95% extended bootstrapped CI < 0 or lower bound 95% extended bootstrapped CI > 0; gray: vehicle, cyan: raclopride). Orange lines indicate a statistically significant difference between the zdF/F_0 of vehicle-treated and SCH23390- or raclopride-treated mice (permutation test). Data are expressed as means ± SEM.

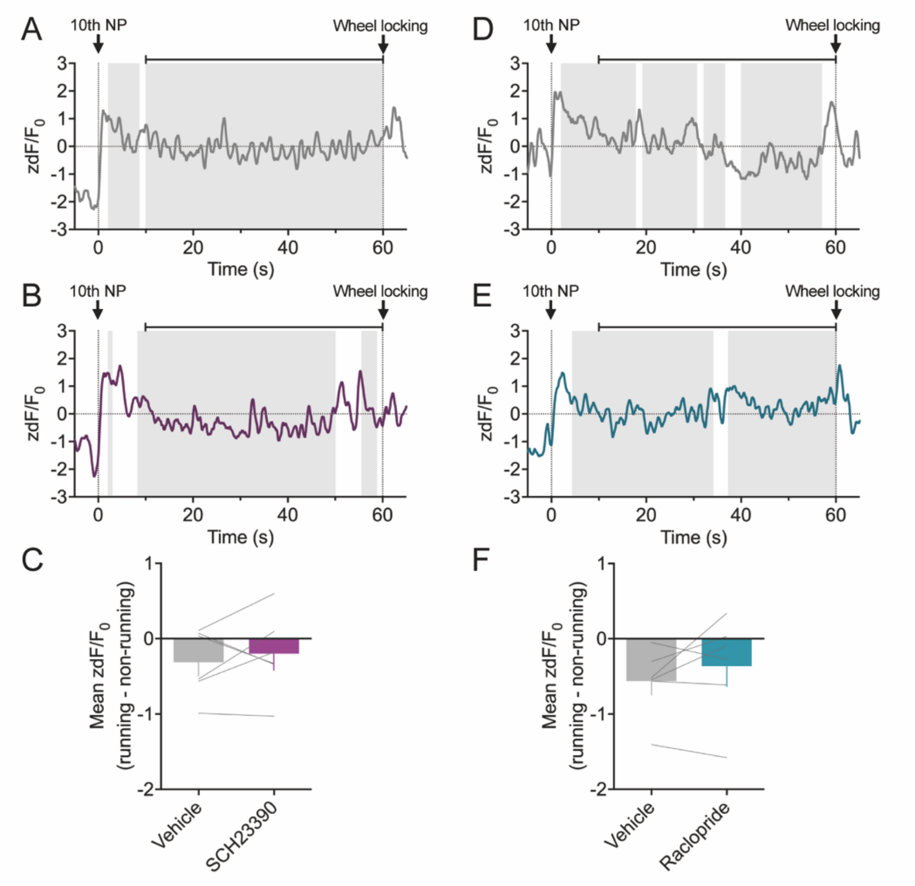

Fig. S9. Effect of systemic dopamine D_1 and D_2 receptor inhibition on mNAc neural activity during consummatory behavior for wheel running. **A, B** Representative GCaMP signals during the wheel-unlocked period following the 10th nose poke in a vehicle- (**A**) and an SCH23390-treated mouse (**B**). Gray shadings indicate periods when the mouse was running on the wheel. Horizontal black line indicates the time window used for analysis. **C** Mean GCaMP signals during wheel running, normalized to non-wheel running periods, in vehicle- and SCH23390-treated mice (n = 6; $t_5 = 0.6253$, $P = 0.5592$, paired *t*-test). **D, E** Representative GCaMP signals during the wheel-unlocked period following the 10th nose poke in a vehicle- (**D**) and a raclopride-treated mouse (**E**). Gray shadings indicate periods when the mouse was running on the wheel. Horizontal black line indicates the time window used for analysis.

F Mean GCaMP signals during wheel running, normalized to non-wheel running periods, in vehicle- and raclopride-treated mice (n = 6; $t_5 = 1.154$, $P = 0.3006$, paired *t*-test).
